# Carnosic Acid and Carnosol Activate AMPK, Suppress Expressions of Gluconeogenic and Lipogenic Genes, and Inhibit Proliferation of HepG2 Cells

**DOI:** 10.3390/ijms22084040

**Published:** 2021-04-14

**Authors:** Shun Hasei, Takeshi Yamamotoya, Yusuke Nakatsu, Yukino Ohata, Shota Itoga, Yuji Nonaka, Yasuka Matsunaga, Hideyuki Sakoda, Midori Fujishiro, Akifumi Kushiyama, Tomoichiro Asano

**Affiliations:** 1 Department of Medical Chemistry, Division of Molecular Medical Science, Graduate School of Biomedical Sciences, Hiroshima University, 1-2-3 Kasumi, Minami-ku, Hiroshima City, Hiroshima 734-8551, Japan; b151007@hiroshima-u.ac.jp (S.H.); ymmty@hiroshima-u.ac.jp (T.Y.); nakatsu@hiroshima-u.ac.jp (Y.N.); sunstone.neige11@gmail.com (Y.O.); 2 Research Institute, Suntory Global Innovation Center Limited, 8-1-1 Seikadai, Seika-cho, Soraku-gun, Kyoto 619-0284, Japan; Shota_Itoga@suntory.co.jp (S.I.); Yuji_Nonaka@suntory.co.jp (Y.N.); 3 Center for Translational Research in Infection & Inflammation, School of Medicine, Tulane University, 6823 St. Charles Avenue, New Orleans, LA 70118, USA; ymatsunaga@tulane.edu; 4 Department of Internal Medicine, Division of Neurology, Respirology, Endocrinology and Metabolism, Faculty of Medicine, University of Miyazaki, Miyazaki 889-1692, Japan; hideyuki_sakoda@med.miyazaki-u.ac.jp; 5 Department of Internal Medicine, Division of Diabetes and Metabolic Diseases, Nihon University School of Medicine, Tokyo 173-8610, Japan; fujishiro.midori@nihon-u.ac.jp; 6 Department of Pharmacotherapy, Meiji Pharmaceutical University, Kiyose City, Tokyo 204-8588, Japan; kushiyama@my-pharm.ac.jp

**Keywords:** carnosic acid, carnosol, rosemary, AMPK, gluconeogenesis, lipogenesis, hepatocellular carcinoma, liver

## Abstract

Carnosic acid (CA), carnosol (CL) and rosmarinic acid (RA), components of the herb rosemary, reportedly exert favorable metabolic actions. This study showed that both CA and CL, but not RA, induce significant phosphorylation of AMP-dependent kinase (AMPK) and its downstream acetyl-CoA carboxylase 1 (ACC1) in HepG2 hepatoma cells. Glucose-6-phosphatase (G6PC) and phosphoenolpyruvate carboxykinase 1 (PCK1), rate-limiting enzymes of hepatic gluconeogenesis, are upregulated by forskolin stimulation, and this upregulation was suppressed when incubated with CA or CL. Similarly, a forskolin-induced increase in CRE transcriptional activity involved in G6PC and PCK1 regulations was also stymied when incubated with CA or CL. In addition, mRNA levels of ACC1, fatty acid synthase (FAS) and sterol regulatory element-binding protein 1c (SREBP-1c) were significantly reduced when incubated with CA or CL. Finally, it was shown that CA and CL suppressed cell proliferation and reduced cell viability, possibly as a result of AMPK activation. These findings raise the possibility that CA and CL exert a protective effect against diabetes and fatty liver disease, as well as subsequent cases of hepatoma.

## 1. Introduction

Patients with metabolic syndromes, including diabetes mellitus, hyperlipidemia, hepatosteatosis and so on, are increasing in number in many countries. While numerous pharmaceutical treatments have been developed and are now available, attention to daily life factors, such as exercise and food intake, remains critical for the prevention of these disorders. Thus, recently, much attention has focused on healthy foods and extracts from natural products. These healthy extracts have different mechanisms of action, including effects on signal transduction, gene transcription, intestinal bacterial flora and so on. Each of these actions is speculated to ameliorate metabolic abnormalities and/or obesity. 

*Rosmarinus officinalis* L. is an herbal plant widely used because of its beneficial health properties. Rosemary extract reportedly contains rosmarinic acid (RA), carnosic acid (CA) and carnosol (CL). While previous reports have suggested potential roles of each of these components in exerting favorable effects, such as enhancement of glucose uptake in skeletal muscle cells [1,2,3] and inhibition of lipid accumulation in adipocytes [4,5], the studies were performed in different cell types and animals. Thus, the molecular mechanism(s) leading to metabolically beneficial consequences may differ depending on the tissue or cell type as well as the individual components of rosemary extract.

AMP-dependent kinase (AMPK) is a serine/threonine kinase that is activated when the intracellular AMP/ATP ratio is high and, therefore, is widely acknowledged to be a cellular energy sensor [6,7]. In general, AMPK activation exerts metabolically beneficial effects. In muscle, for example, AMPK activates glucose uptake independently of insulin and increases mitochondrial biogenesis via activation of peroxisome proliferator-activated receptor (PPAR) γcoactivator 1alpha (PGC-1α). In the liver, AMPK also inhibits de novo lipogenesis via inhibitory phosphorylation of acetyl-CoA carboxylase (ACC), which reduces the synthesis of malonyl CoA. The cellular malonyl CoA level reduction releases the allosteric inhibition of carnitine palmitoyltransferase 1 (CPT1), which consequently enhances fatty acid β-oxidation in mitochondria. AMPK activation was demonstrated to alleviate hepatic steatosis in a murine model [8], and metformin, the most widely used antidiabetic drug among humans, exerts its glucose-lowering effect at least partly by activating AMPK. Therefore, considerable research attention has been focused on finding natural products, such as Rhodiola crenulata, curcumin and clove, capable of activating AMPK [9,10,11].

In this study, we first reconfirmed the effect of AMPK activation by rosemary extract in HepG2 cells [12] and further examined which component(s) of this extract, CA, CL and RA, activate AMPK. Our analysis revealed that both CA and CL, but not RA, activate AMPK in HepG2 cells. We next analyzed the effects of CA and CL on forskolin-induced gluconeogenic gene expressions by employing a quantitative real-time PCR and a luciferase reporter assay. As AMPK plays an important role in suppressing lipogenesis and reducing hepatic lipid accumulation, the effects of lipogenic gene expressions were also investigated. Finally, because AMPK activation reportedly suppresses the growth of cancer cells, the effects of CA and CL on the proliferation of HepG2 cells were investigated. Herein, we present evidence that CA and CL activate AMPK and may exert favorable effects on metabolism and cancer prevention.

## 2. Results

### 2.1. Rosemary Extract and Two of Its Components, CA and CL, Induce AMPK Phosphorylation

**Figure 1:**
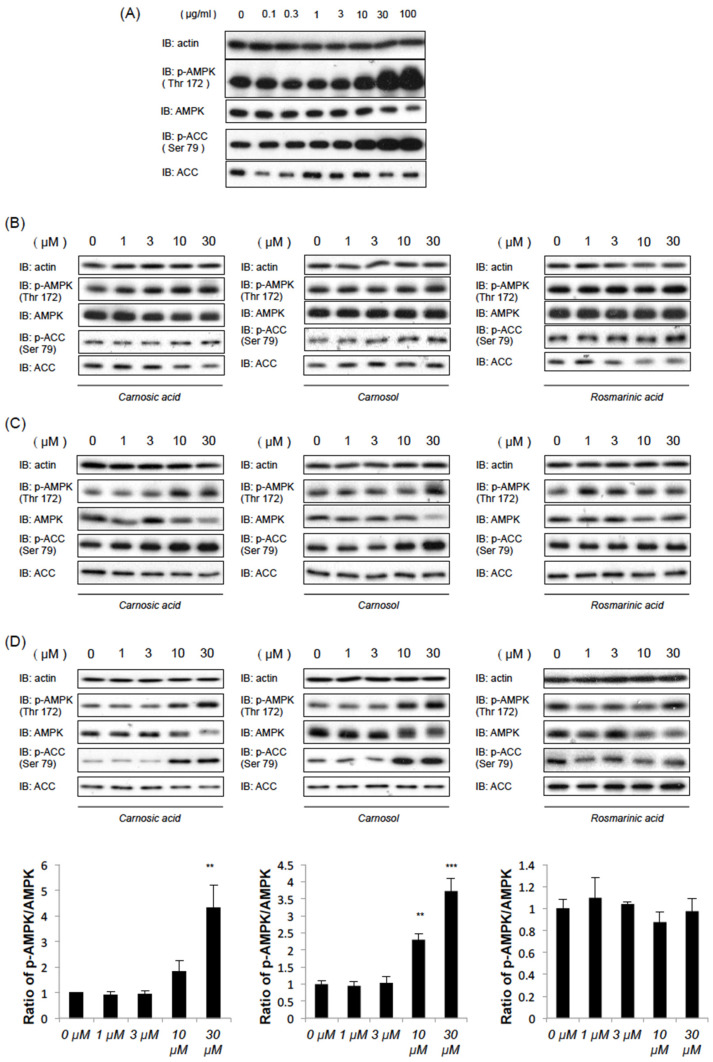
Carnosic acid (CA) and carnosol (CL), but not rosmarinic acid (RA), dose-dependently activate AMP-depenpent kinase (AMPK). After serum starvation for 3 h, the cells were treated with each compound at the indicated concentrations for 1 h. (**A**) The effects of rosemary extract on C2C12 myotubes. (**B**–**D**) The effects of CA, CL and RA in C2C12 myotubes (**B**), HEK-293T (**C**) or HepG2 cells (**D**). Relative band intensities (p-AMPK/AMPK) are also shown in Figure 1D. (**: *p* < 0.01, ***: *p* < 0.001).

We first examined the effects of commercially available rosemary extract on AMP-dependent kinase (AMPK) activation. Exposure of C2C12 myotubes to different concentrations of rosemary extracts (0, 0.1, 0.3, 1, 3, 10, 30, and 100 μg/mL) for 1 h resulted in dose-dependent increases in the phosphorylation of AMPK (Thr 172) and acetyl-CoA carboxylase (ACC) (Ser79), which is well known to be a phosphorylation target site for AMPK (Figure 1A). Next, we examined whether carnosic acid (CA), carnosol (CL) or rosmarinic acid (RA), which are the main components of rosemary extract [13,14], can induce AMPK phosphorylation in HEK-293T cells, C2C12 myotubes and HepG2 cells. As shown in Figure 1B–D, CA and CL concentration-dependently induced AMPK and ACC phosphorylations 1 h poststimulation, while AMPK activation was less noticeable in response to stimulation with RA. As the increase in ACC phosphorylation reached statistical significance at 10 μM for both CA and CL (Figure S1), we examined the effects of CA and CL at the 10 μM concentration in subsequent experiments.

### 2.2. CA and CL Suppress the Induction of Gluconeogenic Genes

**Figure 2:**
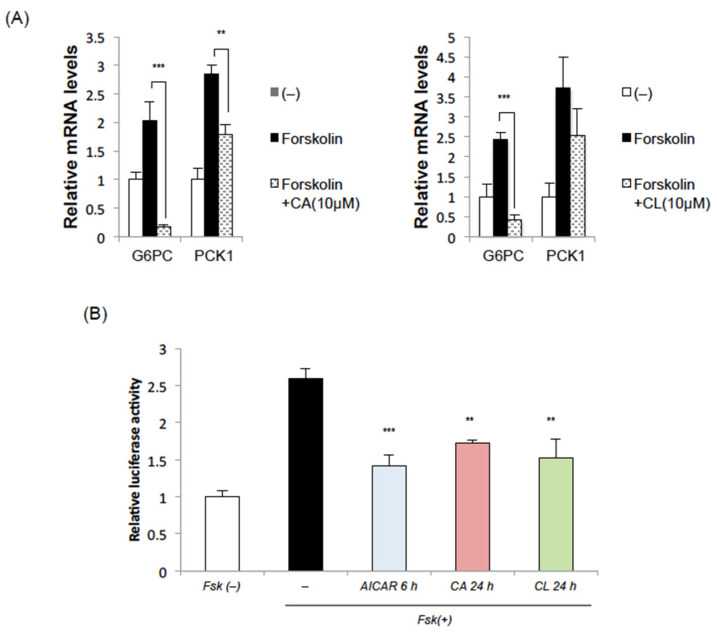
CA and CL suppress gluconeogenic gene induction in HepG2 cells. (**A**) HepG2 cells were treated with CA (10 μM) or CL (10 μM) for 24 h and then with forskolin for 2 h. (**B**) Luciferase assay of PCK1 promoter activity. (**: *p* < 0.01. ***: *p* < 0.001).

Since we previously showed AMPK activation to suppress gluconeogenesis by reducing phosphoenolpyruvate carboxykinase 1 (PCK1) expression in the liver [15], we examined the effects of CA and CL on gluconeogenic gene expressions in the liver. Both CA and CL significantly inhibited the expressions of glucose-6-phosphatase (G6PC) and the PCK1 gene induced by forskolin in HepG2 cells (Figure 2A). Employing the luciferase assay, we found PCK1 promoter activity to be increased by treatment with forskolin, and that this increase was significantly suppressed by pretreatment with AICAR, CA or CL (Figure 2B). These data suggest that CA and CL suppress gluconeogenesis in hepatocytes.

### 2.3. CA and CL Exert Effects on Fatty Acid Synthesis and Oxidation

**Figure 3:**
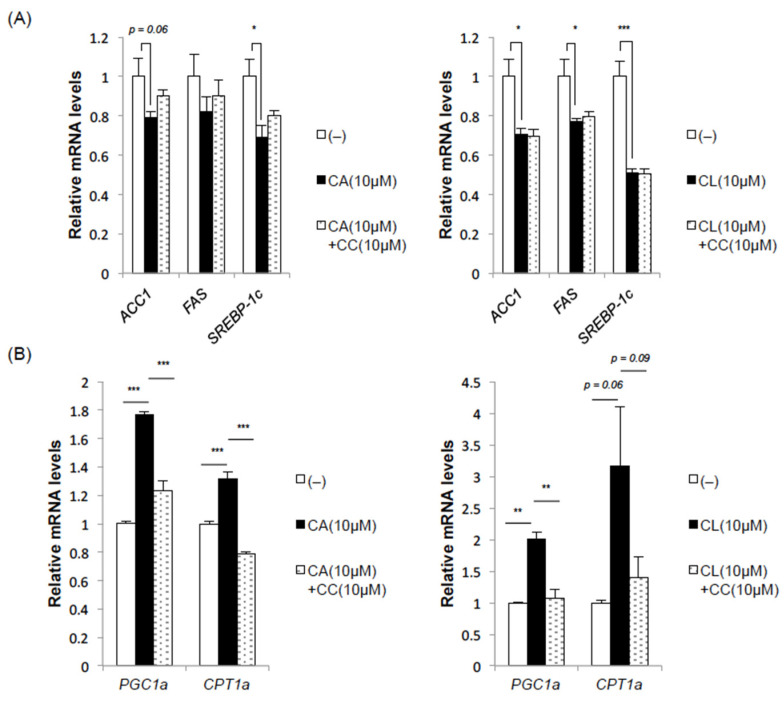
CA and CL suppress lipogenic gene expressions while upregulating genes related to fatty acid oxidation in HepG2 cells. HepG2 cells were pretreated with compound C (CC) (10 μM) for 1 h and then stimulated with CA (10 μM) or CL (10 μM) for 24 h. (**A**) Lipogenic gene expressions. (**B**) Expressions of genes related to fatty acid oxidation (*: *p* < 0.05, **: *p* < 0.01. ***: *p* < 0.001).

Next, we examined the effects of CA and CL on fatty acid synthesis and fatty acid oxidation, which are important factors in the pathogenesis of hepatosteatosis and thereby indirectly affect glucose metabolism. The addition of CA or CL reduced the expression levels of fatty acid synthase (FAS), ACC and sterol regulatory element-binding protein 1c (SREBP-1c), all three of which regulate fatty acid synthesis (Figure 3A). The suppression of these lipogenic gene expressions by CA treatment was partially blocked by pretreatment with the AMPK inhibitor compound C (CC), whereas the suppression by CL was not, suggesting a contribution of AMPK-independent mechanisms at least in CL-mediated suppression of lipogenesis (Figure 3A). 

Interestingly, both CA and CL markedly upregulated peroxisome proliferator-activated receptor (PPAR) γcoactivator-1 alpha (PGC-1α) (Figure 3B), a transcription cofactor that interacts with transcription factors such as PPARα and PPARγ, and has been reported to induce various genes involved in mitochondrial biogenesis and fatty acid oxidation. This upregulation of PGC-1α by CA and CL was completely blocked by pretreatment with CC (Figure 3B), indicating that this upregulation of PGC-1α is AMPK dependent. Consistently, the expression of carnitine palmitoyltransferase 1a (CPT1a), which mediates the transport of acyl-CoA from the cytosol into mitochondria and is therefore crucial for fatty acid oxidation in the liver, was also increased by treatment with CA or CL, and this increase was stymied by pretreatment with CC.

Taken together, these data imply that both CA and CL might, independently of each other, prevent hepatic lipid accumulation by suppressing lipogenesis and enhancing fatty acid oxidation.

### 2.4. CA and CL Inhibit Cell Proliferation and Promote Apoptosis

As has been demonstrated by studying metformin, AMPK is a potential target for cancer prevention and treatment [16]. We thus investigated whether CA or CL could inhibit cell proliferation and/or activate apoptosis in HepG2 cells.

**Figure 4:**
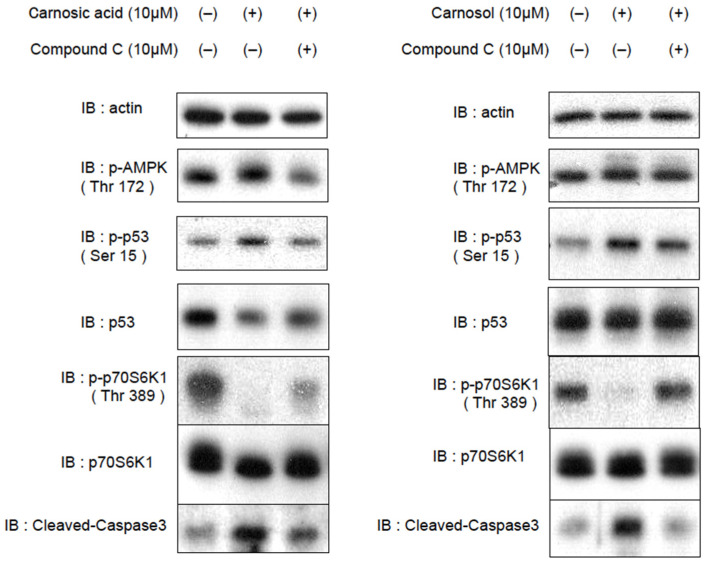
CA and CL activate p53, inhibit the mTORC1-S6K pathway and promote apoptosis in HepG2 cells. After being serum starved for 3 h, HepG2 cells were treated with CA (10 μM) or CL (10 μM), with and without CC (10 μM, 1 h) pretreatment.

To assess direct cellular responses to CA and CL, we treated HepG2 cells with CA or CL, with and without CC pretreatment, and harvested the cells 1 h later. AMPK phosphorylates p53 at Ser15 [17], and this phosphorylation is essential for AMPK-induced cell-cycle arrest [18]. The activation of p53 also induces apoptosis in cancer cells [19]. Furthermore, AMPK phosphorylates TSC2 and Raptor, and inhibits mechanistic target of rapamycin (mTOR) complex 1 (mTORC1), which is often aberrantly activated in cancer cells and therefore a promising target for cancer therapy. In line with AMPK activation, the phosphorylation of p53 and cleaved caspase-3 were increased, and the phosphorylation of p70S6K1 was decreased in response to the addition of CA or CL, but these changes were reversed, at least partially, by pretreatment with CC (Figure 4, Figures S2 and S3).

**Figure 5:**
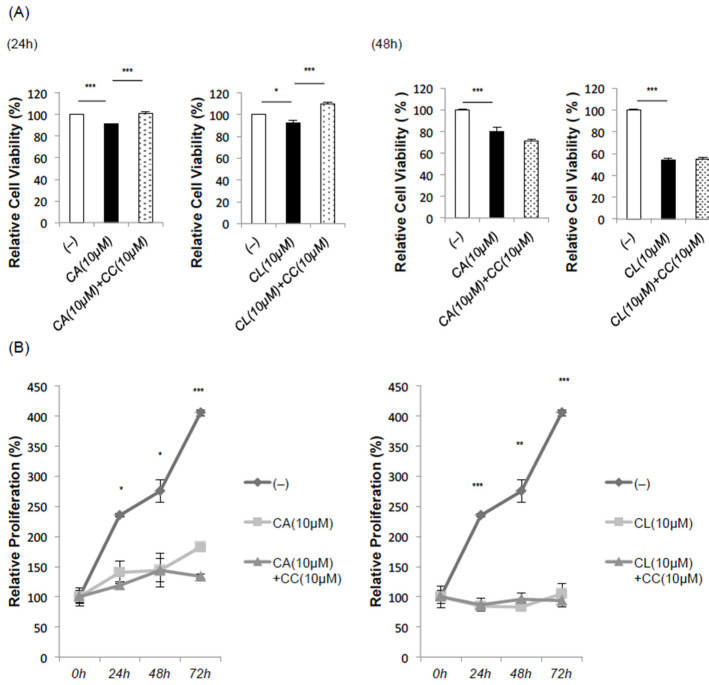
CA and CL inhibit proliferation of HepG2 hepatoma cells. (**A**) MTT assay. After pretreatment with CC (10 μM) for 1 h, HepG2 cells were treated with CA (10 μM) or CL (10 μM) for 24 h (left panel) and 48 h (right panel), and then subjected to MTT assay. (**B**) Cell proliferation assay. (*: *p* < 0.05, **: *p* < 0.01. ***: *p* < 0.001).

Consistently, the methyl thiazolyl tetrazolium (MTT) assay revealed slightly but significantly reduced absorbances 24 h after treatment with CA or CL, reductions which were blocked by CC treatment (Figure 5A). It is noteworthy that the influence of the growth inhibitory effect was weaker in C2C12 fibroblasts (Figure S4). 

However, CC could not reverse the decline in cell viability and/or proliferation when assessed 48 h after the treatment with CA or CL. Furthermore, TdT-mediated digoxygenin-dUTP nick end labeling (TUNEL) staining revealed apoptotic cells when performed 24 h after the treatment with CA or CL, an effect that was not nullified by pretreatment with CC (Figure S5). These results are presumably due to the toxicity of CC itself, since CC reportedly induces apoptosis in cancer cells in an AMPK-independent manner [20]. 

In line with these observations, in the cell proliferation assay, growth inhibition was observed in response to both CA and CL when administered alone, but unlike the MTT assay, no CC-mediated recovery was observed (Figure 5B). 

These data indicate that CA and CL inhibit cancer cell proliferation and promote apoptosis at least partly by activating AMPK.

## 3. Discussion

The liver is the main organ that produces glucose through glycogenolysis and gluconeogenesis in order to sustain blood glucose levels in the fasted state. However, in the presence of insulin resistance, impaired insulin action results in a lack of efficient suppression of gluconeogenesis even in the fed state due to aberrant expressions of gluconeogenic genes, such as glucose-6-phosphatase (G6PC) and phosphoenolpyruvate carboxykinase 1 (PCK1), in the liver as well as an increased supply of gluconeogenic substrates, such as glycerol, to the liver caused by enhanced lipolysis in adipose tissue. Therefore, substances that efficiently inhibit the induction of gluconeogenic gene expressions are potentially promising for the treatment of hyperglycemia. In addition to 7-O-methylrosmanol and royleanonic acid, other rosemary extracts that have been reported to suppress gluconeogenic gene expressions [21], we found that both carnosic acid (CA) and carnosol (CL) also significantly suppress cAMP-induced G6PC and PCK1 expressions, which is in line with our previous study showing that AMP-dependent kinase (AMPK) activation suppresses gluconeogenesis [15]. Interestingly, peroxisome proliferator-activated receptor (PPAR) γcoactivator-1 alpha (PGC-1α) was significantly induced by CA or CL treatment, and these inductions were apparently AMPK dependent. Considering that hepatic expression of PGC-1α correlates negatively with the severity of hepatic steatosis [22], upregulation of PGC-1α and carnitine palmitoyltransferase 1a (CPT1a) by CA or CL treatment suggests enhanced fatty acid oxidation and implies that these compounds would have benefits in alleviating hepatic lipid accumulation. Although it must be noted that PGC-1α activation in the liver reportedly enhances gluconeogenesis by inducing tribbles homolog 3 (TRB-3), an inhibitor of Protein kinase B (Akt) [23], our observations suggest that suppression of gluconeogenic gene expressions via direct activation of AMPK outweighs the indirect effects of PGC-1α induction in CA or CL-treated HepG2 cells.

CA and CL also inhibited the expressions of lipogenic genes such as acetyl-CoA carboxylase 1 (ACC1), fatty acid synthase (FAS) and sterol regulatory element-binding protein 1c (SREBP-1c), but this inhibitory effect seemed to be at least partially AMPK independent. A previous report showed that CA prevented palmitate-induced lipid accumulation in HepG2 cells by decreasing the expression of PPARγ in a manner dependent on the epidermal growth factor receptor/mitogen-activated protein kinase (EGFR/MAPK) pathway but not the AMPK pathway [24]. Therefore, unlike the genes related to fatty acid oxidation, we can reasonably speculate that the downregulation of genes related to lipogenesis from CA or CL treatment is mostly mediated by AMPK-independent mechanisms.

The anticancer activities of rosemary extracts have been intensively studied, as summarized in a recent review [25]. For example, several reports have shown that CA induces apoptosis in HepG2 cells [26,27], but its anticancer activity through AMPK activation has yet to be fully clarified, though CL does inhibit the growth of human prostate cancer PC3 cells at least partly via AMPK activation [28]. We observed that short-term CA or CL treatment in HepG2 cells activated p53 (Figure S2), inhibited mechanistic target of rapamycin (mTOR) complex 1 (mTORC1) and increased caspase-3 cleavage, effects which were all AMPK dependent. However, we also observed that the long-term inhibitory effects of CA or CL on cell proliferation were not nullified by cotreatment with CC, which might be attributable to the anticancer effects of CA or CL that are dependent on mechanisms other than AMPK, as well as the toxicity of CC itself as mentioned above [20]. 

In summary, we have herein shown that CA and CL, components of rosemary extract, attenuate both cAMP-induced gluconeogenic gene expressions and lipogenic gene expressions. In addition, CA and CL induced the expressions of PGC1α and CPT1a, apparently via AMPK activation, suggesting that CA and CL promote fatty acid oxidation in HepG2 cells. Furthermore, CA and CL significantly inhibited the proliferation of HepG2 cells, which was at least partly attributable to their AMPK-activating effects. These findings raise the possibility that CA and CL exert protective effects against diabetes and fatty liver disease, as well as the resultant occurrence of hepatoma.

## 4. Materials and Methods

### 4.1. Reagents

Rosemary extract (RM-21B base) was obtained from Mitsubishi-Chemical Foods Corporation (Tokyo, Japan). The RM-21B base mainly contains water-insoluble components of rosemary extract, such as carnosol (16.8%), carnosic acid (2.8%), rosmanol (3.1%) and epirosmanol (1.3%). Carnosic acid (CA), carnosol (CL) and rosmarinic acid (RA) were purchased from Tokyo Chemical Industry (Tokyo, Japan), Cayman Chemical (Ann Arbor, MI, USA) and FUJIFILM Wako (Osaka, Japan), respectively. Dorsomorphin (compound C) was purchased from Abcam (Cambridge, UK), and AICA riboside (AICAR) from Wako (Osaka, Japan). Purities of the purchased compounds were as follows: CA > 97%, CL > 98%, RA > 96%.

### 4.2. Cell Culture

HepG2 cells, HEK293T cells and C2C12 myoblasts were cultured in Dulbecco’s modified Eagle medium (DMEM) with 10% fetal bovine serum and penicillin–streptomycin (penicillin: 100 U/mL, streptomycin: 100 μg/mL) in a humidified atmosphere of 95% air and 5% CO_2_ at 37 °C. Skeletal muscle differentiation of C2C12 cells was induced by replacing the medium with DMEM supplemented with 2% horse serum for 5–8 days.

### 4.3. Immunoblotting

HepG2 cells were harvested and boiled with a sample buffer (1 × DB). Proteins were separated by SDS-PAGE and transferred to Poly Vinylidene Di-Fluoride (PVDF) membranes. After blocking with 3% bovine serum albumin or 3% skim milk for 1 h, the membranes were incubated with a primary antibody overnight at 4 °C. After being washed with phosphate-buffered saline (PBS) containing 0.1% Tween 20, the membranes were reacted with an antimouse or rabbit IgG horseradish peroxidase-linked secondary antibody (1:10,000) for 1 h at room temperature. Membranes were washed with PBS three times and subjected to immunoblotting using SuperSignal West Pico Substrate (Thermo Scientific, Waltham, MA, USA) or ImmunoStar LD (Wako). Band intensities were quantitatively analyzed using ImageJ. The following antibodies were used: actin (sc-47778, Santa Cruz Biotechnology, Dallas, TX, USA), AMPK (#5832, Cell Signaling Technology, Danvers, MA, USA), p-AMPK (#2535, Cell Signaling Technology), ACC (#3662, Cell Signaling Technology), p-ACC (#11818, Cell Signaling Technology), p53 (sc-393031, Santa Cruz Biotechnology), p-p53 (#9284, Cell Signaling Technology), p70S6K1 (#2708, Cell Signaling Technology), p-p70S6K1 (#9234, Cell Signaling Technology), cleaved caspase-3 (#9664, Cell Signaling Technology), caspase-3 (#9665, Cell Signaling Technology).

### 4.4. Quantitative Real-Time PCR

Total RNAs from cells were isolated using Sepasol reagent (Nakalai Tesque, Kyoto, Japan), according to the manufacturer’s instructions. Template cDNA was synthesized from total RNA using Verso cDNA synthesis kits (Thermo Fisher Scientific K.K.). Quantitative real-time PCR was performed using the CFX96 real-time PCR system (Bio-Rad, Hercules, CA, USA) with SYBR Green (Agilent Technology, Santa Clara, CA, USA).

The following primers were designed: GAPDH: forward, 5’-GGCCTCCAAGGAGTAAGACC-3’, reverse, 5’-AGGGGTCTACATGGCAACTG-3’; G6Pase: forward, 5’-GGCTCAACCTCGTCTTTAAGTG-3’, reverse, 5’-CTCCCTGGTCCAGTCTCACA-3’; PCK1: forward, 5’-ACGGATTCACCCTACGTGGT-3’, reverse, 5’-CCCCACAGAATGGAGGCATTT-3’; ACC1: forward, 5’-ATCCCAGCTGATCCAGCAAA-3’, reverse, 5’-ACTCCCTCAAGCCATCCACA-3’; FAS: forward, 5’-AACTTGGAAGGCCTGCATCA-3’, reverse, 5’-CCGGTGCAGTTTATTTCCA-3’; SREBP-1c: forward, 5’-TCTCTACAGGAAGCCCTCCC-3’, reverse, 5’-CCGTCTGTCTTCATGGCTGT-3’; CPT1a: forward, 5’-GATTTTGCTGTCGGTCTTGG-3’, reverse, 5’-CTCTTGCTGCCTGAATGTGA-3’.

### 4.5. Luciferase Assay

HepG2 cells were cotransfected with a PCK1-Luc vector, which is a PCK1 promoter (–450 to –1) inserted into a pGL4 [10], and a TK-Rluc vector using Lipofectamine 3000 (Invitrogen). The cells were treated with AICAR (2 mM), CA (10 μM) or CL (10 μM) for the indicated times under serum-free conditions with or without forskolin (10 μM) for the last 6 h before harvesting. Luciferase activities were measured using a Dual-Glo Luciferase Assay System (Promega, Madison, WI, USA), and PCK1 promoter activities were expressed as firefly luciferase activities after being normalized by the corresponding Renilla luciferase activities.

### 4.6. Cell Viability

Cell viability was determined by MTT assay. The MTT reagent was prepared by dissolving 3-(4,5-dimethylthiazol-2-yl)-2,5-diphenyltetrazolium bromide (Sigma-Aldrich, St. Louis, MO, USA) at a concentration of 2.5 mg/mL in sterilized water. HepG2 cells were cultivated at a concentration of 1 × 10^5^ cells/well in a 24-well plate. After pretreatment with compound C (CC) for 1 h, CA or CL was added to the culture medium at the indicated concentrations for 24 or 48 h. The MTT reagent was added to each well, and, after incubation for 3 h, absorbance at 570 nm was measured (TriStar LB941 Multimode Microplate Reader, BERTHOLD TECHNOLOGIES GmbH&Co. KG, Bad Wildbad, Germany).

### 4.7. Cell Proliferation Assay

HepG2 cells were plated onto 24-well plates (1.5 × 10^5^ cells/well) and incubated overnight. CC was added 1 h before the treatments with CA or CL. After the addition of either CA or CL, the cells were trypsinized, and cell numbers were manually counted using a hemocytometer at the indicated times. 

### 4.8. Statistical Analysis

Analysis of variance (ANOVA) as well as Tukey’s and Dunnett’s post-hoc tests were used to determine the significance of the differences between groups. EZR (Saitama Medical Center, Jichi Medical University, Saitama, Japan) was used for all calculations. We considered *p* < 0.05 to indicate a statistically significant difference. The *p* values are presented as follows: *p* < 0.05 (*), *p* < 0.01 (**), or *p* < 0.001 (***).

## Data Availability

The data presented in this study are available on request from the corresponding authors.

## Supplementary Materials

The following are available online at https://www.mdpi.com/article/10.3390/ijms22084040/s1.

## References

[1] Naimi M., Vlavcheski F., Murphy B., Hudlicky T., Tsiani E. (2017). Carnosic acid as a component of rosemary extract stimulates skeletal muscle cell glucose uptake via AMPK activation. Clin. Exp. Pharmacol. Physiol..

[2] Vlavcheski F., Naimi M., Murphy B., Hudlicky T., Tsiani E. (2017). Rosmarinic Acid, a Rosemary Extract Polyphenol, Increases Skeletal Muscle Cell Glucose Uptake and Activates AMPK. Molecules.

[3] Vlavcheski F., Baron D., Vlachogiannis I.A., MacPherson R.E.K., Tsiani E. (2018). Carnosol Increases Skeletal Muscle Cell Glucose Uptake via AMPK-Dependent GLUT4 Glucose Transporter Translocation. Int. J. Mol. Sci..

[4] Park M.Y., Sung M.K. (2015). Carnosic Acid Inhibits Lipid Accumulation in 3T3-L1 Adipocytes Through Attenuation of Fatty Acid Desaturation. J. Cancer Prev..

[5] Lee Y.H., Lim W., Sung M.K. (2018). Carnosic Acid Modulates Increased Hepatic Lipogenesis and Adipocytes Differentiation in Ovariectomized Mice Fed Normal or High-Fat Diets. Nutrients.

[6] Zhang B.B., Zhou G., Li C. (2009). AMPK: An emerging drug target for diabetes and the metabolic syndrome. Cell Metab..

[7] Mihaylova M.M., Shaw R.J. (2011). The AMPK signaling pathway coordinates cell growth, autophagy and metabolism. Nat. Cell Biol..

[8] Boudaba N., Marion A., Huet C., Pierre R., Viollet B., Foretz M. (2018). AMPK Re-Activation Suppresses Hepatic Steatosis but its Downregulation Does Not Promote Fatty Liver Development. EBioMedicine.

[9] Lee S.-Y., Lai F.-Y., Shi L.-S., Chou Y.-C., Yen I.-C., Chang T.-C. (2015). Rhodiola crenulata extract suppresses hepatic gluconeogenesis via activation of the AMPK pathway. Phytomedicine.

[10] Kang O.H., Kim S.B., Seo Y.S., Joung D.K., Mun S.H., Choi J.G., Lee Y.M., Kang D.G., Lee H.S., Kwon D.Y. (2013). Curcumin decreeses oleic acid-induced lipid accumulation via AMPK phosphorylation in heaptocarcinoma cells. Eur. Rev. Med. Pharmacol. Sci..

[11] Li C., Xu H., Chen X., Chen J., Li X., Qiao G., Tian Y., Yuan R., Su S., Liu X. (2019). Aqueous extract of clove inhibits tumor growth by inducing autophagy through AMPK/ULK pathway. Phytother. Res..

[12] Tu Z., Moss-Pierce T., Ford P., Jiang T.A. (2013). Rosemary (*Rosmarinus officinalis L.*) extract regulates glucose and lipid metabolism by activating AMPK and PPAR pathways in HepG2 cells. J. Agric. Food Chem..

[13] Bai N., He K., Roller M., Lai C.S., Shao X., Pan M.H., Ho C.T. (2010). Flavonoids and phenolic compounds from Rosmarinus officinalis. J. Agric. Food Chem..

[14] Nakagawa S., Greg G.H., Gabriel N. (2020). *Rosmarinus officinalis L*. (Rosemary) Extracts Containing Carnosic Acid and Carnosol are Potent Quorum Sensing Inhibitors of *Staphylococcus aureus* Virulence. Antibiotics.

[15] Horike N., Sakoda H., Kushiyama A., Ono H., Fujishiro M., Kamata H., Nishiyama K., Uchijima Y., Kurihara Y., Kurihara H. (2008). AMP-activated protein kinase activation increases phosphorylation of glycogen synthase kinase 3beta and thereby reduces cAMP-responsive element transcriptional activity and phosphoenolpuruvate carboxykinase C gene expression in the liver. J. Biol. Chem..

[16] Li W., Saud M.S., Young R.M., Chen G., Hua B. (2015). Targeting AMPK for cancer prevention and treatment. Oncotarget.

[17] Imamura K., Ogura T., Kishimoto A., Kaminishi M., Esumi H. (2001). Cell cycle regulation via p53 phosphorylation by a 5’-AMP activated protein kinase activator, 5-aminoimidazole-4-carboxamide-1-beta-D-ribofuranoside, in a human hepatocellular carcinoma cell line. Biochem. Biophys. Res. Commun..

[18] Jones R.G., Plas D.R., Kubek S., Buzzai M., Mu J., Xu Y., Birnbaum M.J., Thompson C.B. (2005). AMP-activated protein kinase induces a p53-dependent metabolic checkpoint. Mol. Cell.

[19] Aubrey B.J., Kelly G.L., Janic A., Herold M.J., Strasser A. (2018). How does p53 induce apoptosis and how does this relate to p53-mediated tumor suppression?. Cell Death Differ..

[20] Dai R.Y., Zhao X.F., Li J.J., Chen R., Luo Z.L., Yu L.X., Chen S.K., Zhang C.Y., Duan C.Y., Liu Y.P. (2013). Implication of transcriptional repression in compound C-induced apoptosis in cancer cells. Cell Death Dis..

[21] Yun Y.S., Noda S., Shigemori G., Kuriyama R., Takahashi S., Umemura M., Takahashi Y., Inoue H. (2013). Phenolic diterpenes from rosemary suppress cAMP responsiveness of gluconeogenic gene promoters. Phytother. Res..

[22] Piccinin E., Villani G., Moschetta A. (2019). Metabolic aspects in NAFLD, NASH and hepatocellular carcinoma: The role of PGC1 coactivators. Nat. Rev. Gastroenterol. Hepatol..

[23] Koo S.H., Satoh H., Herzig S., Lee C.H., Hedrick S., Kulkarni R., Evans R.M., Olefsky J., Montminy M. (2004). PGC-1 promotes insulin resistance in liver through PPAR-alpha-dependent induction of TRB-3. Nat. Med..

[24] Wang T., Takikawa Y., Tabuchi T., Satoh T., Kosaka K., Suzuki K. (2012). Carnosic acid (CA) prevents lipid accumulation in hepatocytes through the EGFR/MAPK pathway. J. Gastroenterol..

[25] Allegra A., Tonacci A., Pioggia G., Musolino C., Gangemi S. (2020). Anticancer Activity of *Rosmarinus officinalis* L.: Mechanisms of Action and Therapeutic Potentials. Nutrients.

[26] Zhang X., Chen Y., Cai G., Li X., Wang D. (2017). Carnosic acid induces apoptosis of hepatocellular carcinoma cells via ROS-mediated mitochondrial pathway. Chem. Biol. Interact..

[27] Xiang Q., Ma Y., Dong J., Shen R. (2015). Carnosic acid induces apoptosis associated with mitochondrial dysfunction and Akt inactivation in HepG2 cells. Int. J. Food Sci. Nutr..

[28] Johnson J.J., Syed D.N., Heren C.R., Suh Y., Adhami V.M., Mukhtar H. (2008). Carnosol, a dietary diterpene, displays growth inhibitory effects in human prostate cancer PC3 cells leading to G2-phase cell cycle arrest and targets the 5’-AMP-activated protein kinase (AMPK) pathway. Pharm. Res..

