# Supplementary material for: Carnosic Acid and Carnosol Activate AMPK, Suppress Expressions of Gluconeogenic and Lipogenic Genes, and Inhibit Proliferation of HepG2 Cells
Source: Int J Mol Sci. 2021 Apr 14;22(8):4040. doi: 10.3390/ijms22084040 (PMC8070802; doi:10.3390/ijms22084040)
Supplement: Supplementary file 1 [file ijms-22-04040-s001.zip › 3-ijms-1155842-supple.docx]

**Supplementary Materials:** The following are available online at www.mdpi.com/xxx/s1, Figure S1: title, Table S1: title, Video S1: title.

***Fig S1.*** CA and CL dose-dependently activate ACC (Ser79) phosphorylation.

After serum-starvation for 3 h, HepG2 cell was treated with each compound at the indicated concentrations for 1h. (**: P < 0.01, ***: P < 0.001)

***Fig S2.*** Quantitative analysis of p53 phosphorylation when stimulated with CA or CL.

After serum-starvation for 3 h and pre-incubation with CC for 1 h, HepG2 cells were treated with each compound for 1 h. The relative band intensities of p-p53 (adjusted by p53) are shown. (***: P < 0.001)

***Fig S3.*** CA and CL induce caspase3 cleavage at the 10 μM concentration.

After serum-starvation for 3 h, HepG2 cells were treated with each compound for 1 h at the indicated concentration for 1 h.

***Fig S4.*** C2C12 myoblasts are less prone to growth inhibition by CA or CL.

MTT assay. After pre-incubation with CC (10 μM) for 1 h, C2C12 cells were treated with CA (10 μM) or CL (10 μM) for 24 h and then subjected to MTT assay. (***: P < 0.001)

***Fig S5.*** TUNEL staining in CA or CL-treated HepG2 cells.

　After being pre-incubated with CC for 1 h, HepG2 cells were treated with or without CA or CL for 24 h and then subjected to TUNEL staining. White arrows: TUNEL-positive cells.

**Supplementary Method**

*TUNEL staining*

HepG2 cells were plated onto slides with 8 wells per chamber (3.0×10^4^ cells/well) and incubated overnight. After all of the treatments, the slides were immersed in 4% formaldehyde in PBS. After being washed with PBS, the cells were permeabilized with Triton X-100 and washed with PBS. TUNEL staining was performed using *DeadEnd™ Fluorometric TUNEL System* (Promega) according to the manufacturer’s instruction.
